# 13C-Metabolic Flux Analysis in Developing Flax (*Linum usitatissinum* L.) Embryos to Understand Storage Lipid Biosynthesis

**DOI:** 10.3390/metabo10010014

**Published:** 2019-12-24

**Authors:** Sébastien Acket, Anthony Degournay, Yannick Rossez, Stéphane Mottelet, Pierre Villon, Adrian Troncoso-Ponce, Brigitte Thomasset

**Affiliations:** 1 Alliance Sorbonne Université, Université de Technologie de Compiègne, 60205 Compiègne CEDEX, France; anthony.degournay@utc.fr (A.D.); yannick.rossez@utc.fr (Y.R.); adrian.troncoso-ponce@utc.fr (A.T.-P.); brigitte.thomasset@utc.fr (B.T.); 2 Alliance Sorbonne Université, EA 4297 TIMR, Transformations Intégrées de la Matière Renouvelable, Université de Technologie de Compiègne, 60205 Compiègne CEDEX, France; stephane.mottelet@utc.fr; 3 Alliance Sorbonne Université, Laboratoire Roberval, FRE UTC CNRS 2012, Université de Technologie de Compiègne, 60205 Compiègne CEDEX, France; pierre.villon@utc.fr

**Keywords:** parallel labeling experiment, metabolic flux analysis, flax, embryo

## Abstract

Flax (*Linum usitatissinum* L.) oil is an important source of α-linolenic (C18:3 ω-3). This polyunsaturated fatty acid is well known for its nutritional role in human and animal diets. Understanding storage lipid biosynthesis in developing flax embryos can lead to an increase in seed yield via marker-assisted selection. While a tremendous amount of work has been done on different plant species to highlight their metabolism during embryo development, a comprehensive analysis of metabolic flux in flax is still lacking. In this context, we have utilized in vitro cultured developing embryos of flax and determined net fluxes by performing three complementary parallel labeling experiments with 13C-labeled glucose and glutamine. Metabolic fluxes were estimated by computer-aided modeling of the central metabolic network including 11 cofactors of 118 reactions of the central metabolism and 12 pseudo-fluxes. A focus on lipid storage biosynthesis and the associated pathways was done in comparison with rapeseed, arabidopsis, maize and sunflower embryos. In our hands, glucose was determined to be the main source of carbon in flax embryos, leading to the conversion of phosphoenolpyruvate to pyruvate. The oxidative pentose phosphate pathway (OPPP) was identified as the producer of NADPH for fatty acid biosynthesis. Overall, the use of 13C-metabolic flux analysis provided new insights into the flax embryo metabolic processes involved in storage lipid biosynthesis. The elucidation of the metabolic network of this important crop plant reinforces the relevance of the application of this technique to the analysis of complex plant metabolic systems.

## 1. Introduction

During the last decades the production and consumption of plant oil-derived products has steadily increased worldwide at a rate of roughly 5% per year [1]. It represents a current market value of over US$120 billion and its consumption is expected to almost double by 2030 [2]. This phenomenon is explained by factors ranging from the exponential growth of the world population and its concomitant increase in food demand to the use of vegetal oils as feedstock for biodiesel production and other industrial products [3,4]. Four crops mainly fulfill this global demand: Oil palm, soybean, rapeseed and sunflower [4,5]. The oil (TAG, triacylglycerols) produced by these plants is stored in different tissues. These oils present qualitative differences in their composition: Palmitic acid (16:0), a saturated fatty acid, is the major fatty acid in palm oil. In contrast, unsaturated fatty acids, e.g., oleic (18:1) and linoleic (18:2) acids [6,7] are well represented in soybean, rapeseed and sunflower. The consumption of these different oils has many repercussions on human health [8,9]. The proportion of the essential fatty acids is important; linoleic (C18:2 ω-6) and α-linolenic (C18:3 ω-3) are both precursors of very-long-chain polyunsaturated fatty acids (VLCPUFA), which are critical for maintaining a healthy physiological condition [8,9]. The ratio ω-6/ω-3 plays a fundamental role in human health. The optimal relationship between both of these unsaturated fatty acids has been identified to be approximately around 4 to 1. However, this ratio is typically higher in current Western diets (10:1 to 25:1). As such, increasing ω-3 intake could improve this balance [10]. Flax oil is an important source of ω-3 fatty acids; α-linolenic acid comprises more than 60% of total oil, with oil representing up to 43% of total seed weight [11,12]. In order to select cultivars richer in ω-3 fatty acids, understanding the carbon sources and metabolic fluxes leading to the synthesis and accumulation of these molecules is a critical step [13,14].

Fatty acid synthesis takes place in the plastids of developing seeds. This process relies on the photosynthetically assimilated carbon that is subsequently exported to the seeds in the form of sucrose [15,16,17]. Within the embryo, sucrose is enzymatically hydrolyzed to fructose and glucose. These hexoses and hexose-phosphate derivatives enter the glycolytic and the oxidative-pentose pathways to generate acetyl-CoA and reduce equivalents. These produce energy for de novo intraplastidial fatty acid synthesis. Glycolysis is compartmentalized within the cytosol and plastids, but both metabolic routes communicate with each other via membrane transporters [17,18,19]. Acetyl-CoA must be synthetized within plastids; to date, the transport of acetyl-CoA across the plastidial membrane has not been demonstrated. Therefore, its glycolytic precursors are either generated within plastids or imported from the cytosol through the action of dedicated, yet to be identified transporters [20]. Because of this dual location, the predominant source of carbon for plastidial acetyl-CoA originates from one of the two glycolytic pathways. While different approaches using diverse plants and tissues have been evaluated in order to identify the main route from sucrose to fatty acids [21,22,23], the complexity of the process and the technical limitations of the methods used make this task difficult. Recently, the importance of the plastidial route during the fatty acid synthesis in *Arabidopsis thaliana* has been described [24].

In the present study, we have identified the major route of intracellular carbon with 13C metabolic flux analysis (13C-MFA) in developing flax embryos. We have obtained the metabolic map by using complementary parallel labeling experiments. The main source of carbon for plastidial fatty acid synthesis in flax embryos is discussed in comparison with 13C-MFA studies of other vegetable crop species [25,26,27,28,29,30]. The recognition of similarities and differences between diverse oilseeds sheds light on the participation of the different carbon fluxes, from sucrose to fatty acids, during the active period of lipid biosynthesis and accumulation of storage lipids in developing oilseeds.

## 2. Results

### 2.1. Optimal Growth Conditions

**Figure 1:**
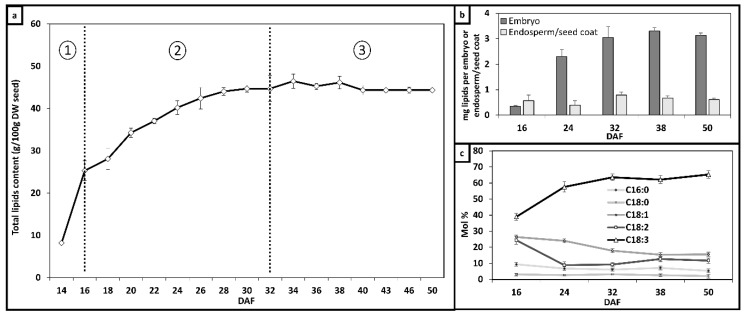
Synthesis of lipids and fatty acids in flax embryos and endosperm/seed coat during seed maturation. (**a**) Total lipid content in flax expressed in g/100 g Dry Weight (DW); (**b**) evolution of total lipids per tissue (mg lipids per embryo or endosperm/seed coat); (**c**) relative fatty acid abundance in embryos during lipid accumulation (mol%). Data is represented as mean and variation (*n* = 4).

To characterize the synthesis of lipids in flax and identify the tissues (embryo versus endosperm/seed coat) that are the primary contributor to lipid flax seed lipid synthesis, lipid accumulation throughout seed development was measured (Figure 1). Capsules were harvested from 14 to 50 DAF (days after flowering), flash frozen in liquid nitrogen and lyophilized. Total lipid content was determined following the protocol described by Bénard et al. (2018) [31]. Lipid content in flax showed significant differences from 14 to 32 DAF (*p*-value < 0.05) (Figure 1a).

Three distinct phases of lipid accumulation were identified during flax embryo development based on differences in the linear velocities of lipid accumulation. At 14–16 DAF (first stage), the rate of lipid synthesis rate was 0.1 mg d^−1^ per seed (R^2^ = 0.99) corresponding to the beginning of lipid synthesis. Between 16–32 DAF (second stage) the highest rate of fatty acid synthesis was attained, with a value of 0.2 mg d^−1^ per seed (R^2^ = 0.97). At 32 DAF (third stage), the oil synthesis rate remained stable (0 mg d^−1^ per seed, R^2^ = 0.99). In flax, the endosperm is a thin layer of cells attached to the internal seed coat (Figure 1b).

The seed coat and endosperm are indistinguishable. Embryos showed a higher content of lipids and rate of synthesis, measured from 16 to 32 DAF, than the endosperm/seed coat: 3.05 mg/embryo and 0.17 mg/d per tissue (R^2^ = 0.99) in the former tissue and 0.62 mg of endosperm/seed coat and 0.01 mg/d per tissue (R^2^ = 0.99) in the latter one. The fatty acid profile of embryos at 16, 24, 32, 38 and 50 DAF were analyzed by GC-MS (Figure 1c). A total of five fatty acids, C16:0, C18:0, C18:1, C18:2 and C18:3, were found in flax embryos. During the middle phase, C18:3 content increased in conjunction with a reduction of C18:2, from 39.9 to 63.5 and from 24.4 to 9.3 mol%, respectively. Low levels of C18:0 and C16:0 remained stable. At 50 DAF, embryo oil was comprised of C16:0 (5.3 mol%), C18:0 (2.1 mol%), C18:1 (15.6 mol%), C18:2 (11.7 mol%) and C18:3 (65.3 mol%).

### 2.2. In Vitro Culture of Flax Embryos

**Figure 2:**
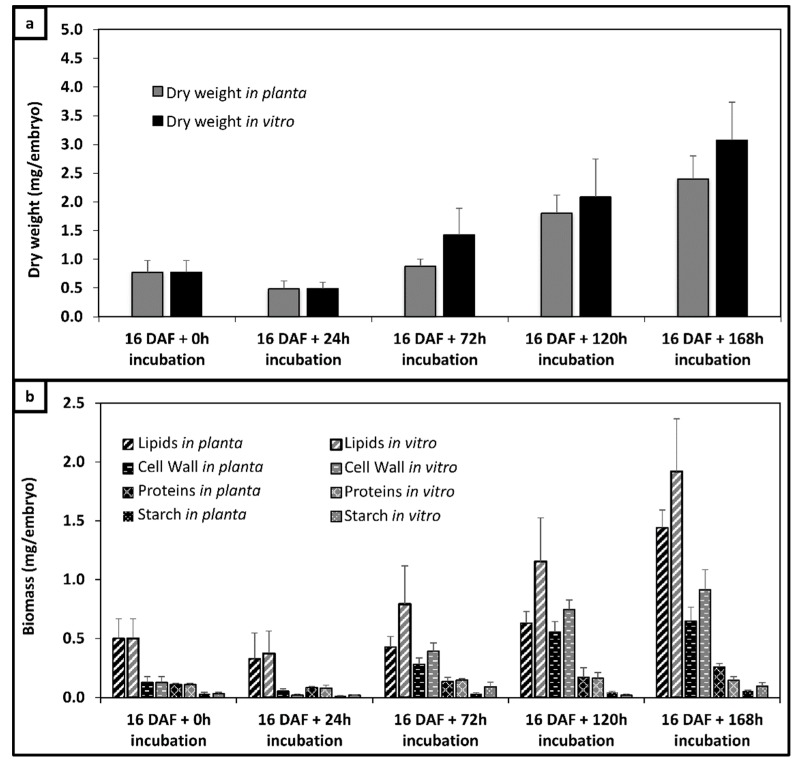
Weight and biomass composition between embryos cultured in vitro and the corresponding state in planta. (**a**) Dry weight between in planta and in vitro expressed in mg/embryo; (**b**) biomass accumulation between in vitro and in planta expressed in mg/embryo. Dry weight and biomass composition (lipids, proteins, cell wall and starch) content were determined as described in the “Materials and Methods” section from flax embryos cultivated from 16 days after flowering (DAF) for 24, 72, 120 and 168 h in culture medium A, containing unlabeled glucose and glutamine as the carbon source. For each kinetic point, four independent experiments were made.

Flax seeds harvested at 16 DAF were dissected and their embryos incubated during 24, 72, 120 and 168 h with unlabeled substrates (culture A). Both in vitro and in planta e < grown embryos showed similar dry weights throughout development, from 0.77 to 3.08 mg/DW and to 2.40 mg/DW, respectively (Figure 2a).

Embryo biomass quantification was performed as previously described [26,31,32]. The main four components constituting 90% of the biomass were mainly lipids (58 ± 2.1%), soluble proteins (22 ± 1.2%), cell walls (11 ± 1.3%) and starch (5 ± 1.5%) in planta. The composition in vitro and in planta during development was statistically similar and in both cases growth rate became linear at 72 h (Figure 2b). Together these results indicate that the in vitro culture conditions provide a good model for studying embryo metabolism.

Concentrations of lipids, proteins, starch and cell wall were used for calculating both lower and upper bounds for external fluxes. External fluxes were calculated from the growth rate of the embryos during their incubation and the biochemical composition of the reserve compounds because the proportion of an embryo’s biochemical components does not change during incubation. These fluxes correspond to the rate of accumulation of lipids (Vtag: 46.21 ± 5 mmol/gDW/d, proteins, Vprot: 105.59 ± 1.5 mmol/gDW/d cell wall, Vcw: 44.4 ± 2.30 mmol/gDW/d and starch, Vsta: 1.0 ± 0.2 mmol/gDW/d). The two main reactions in the 13C-MFA models are TAG and protein synthesis. These reactions utilize the precursor metabolites and cofactors needed for cell expansion.

To determine the coefficients in these reactions, biomass compositions were determined experimentally. Fatty acid composition is presented in Figure 1c and amino acid composition is presented in Supplementary Table S1.

### 2.3. Metabolic and Isotopic Steady State

**Figure 3:**
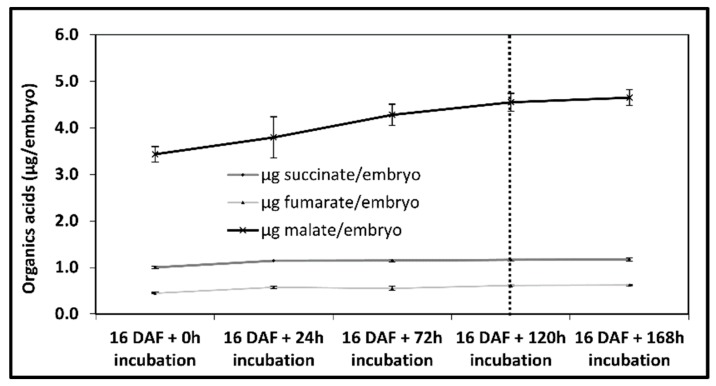
Concentration of organic acids (µg/embryo) during in vitro incubation. Organic acid concentrations were determined as described in the “Materials and Methods” section from flax embryos cultivated from 16 DAF for 24, 72, 120 and 168 h in culture medium A, containing unlabeled glucose and glutamine as the carbon source. For each kinetic point, four independent experiments were carried out.

A 13C-based metabolic flux analysis performed at quasi-stationary steady state requires both metabolic and isotopic steady state conditions. Metabolic steady state is reached when the concentration of metabolic intermediates does not change while there is synthesis of reserve compounds [33]. In the model described in this manuscript, organic acids have been considered as metabolic intermediates and their concentrations have been measured within embryos (16 DAF) incubated for 24, 72, 120 and 168 h in culture medium A. The concentrations of fumarate and succinate increased until reaching 0.45 and 1.15 µg/embryo, respectively, at 72 h of incubation. Malate concentration increased to 4.55 µg/embryo after 120 h of incubation (Figure 3). After 120 h, the concentrations of these intermediates became stable (*p* > 0.05).

With the aim of identifying the time required to reach the isotopic steady state condition, 16 DAF embryos were incubated with 20 mM [U-13C6] glucose, 80 mM glucose unlabeled, 160 mg/L, [U-13C5] glutamine and glutamine unlabeled (culture B).

Isotopic steady state of free sugars [34] and organic acids were obtained at 72 h. Free amino acids [32] and fatty acids required 120 h of incubation (Supplementary Table S2); consequently, the conditions necessary for a quasi-stationary steady state, a metabolic and isotopic steady state, were reached upon 120 h of incubation.

### 2.4. Metabolic Model Construction

The central carbon network model required for 13C-MFA calculations was constructed based on the metabolic information available from different sources, including proteomic studies [21,22], genome sequencing information [35], EST libraries [11,36,37], and other seed-related-data [23,26,27,28,29,30]. The model developed takes into account the stoichiometry of the reactions and describes the central carbon metabolism in developing flax embryos during the lipid accumulation with condition-specific growth stoichiometries based on the measured biomass compositions. The model relies on the breakdown and transformation of two extracellular sources of carbon nutriments, glucose and glutamine; therefore, it considers two input fluxes (Vg and Va, respectively). Similarly, the model also considers five external fluxes corresponding to the accumulation of the main components of the generated biomass during the maturation stage: Starch (Vsta), cell walls (Vcw), CO_2_ output (VCO_2_), triglycerides (Vtag) and proteins (Vprot). These input and external fluxes have been determined experimentally. The model includes all central carbon pathways found in flax embryos, cytosolic and plastidial glycolysis reactions, pentose phosphate pathways, Krebs cycles, anaplerotic and malic enzymes, amino acids, sugars, fatty acid synthesis, cofactor balance, uridine diphosphate kinase (Vnrj4), non-growth associated ATP maintenance (Vnrj5) and ATP synthase. Starch degradation reaction (Vamy1) and ribulose-1,5-bis-phosphate carboxylase reaction (Vrubisco) was included, although flax embryos are heterotrophic during the maturation phase [38]. Cofactors (ATP, ADP, NADP, NADPH, AMP, NAD, NADH, UDP, UTP, FADH2 and FAD) play a paramount role during the synthesis of biomass components; therefore, they have been considered as internal metabolites within the network. The metabolic network is constrained with the carbon balance, the redox and the energy status. A total of 118 reactions of the central carbon metabolism and 12 pseudo-fluxes corresponding to a fraction of TAG and starch, already present in the cell, were modeled. A schematic representation of the model is given in Supplementary Figure S1 and the complete list of reactions and metabolites are listed, respectively, in Supplementary Tables S3 and S4.

### 2.5. 13C Metabolic Flux Analysis

#### 2.5.1. Source of Carbon for Fatty Acids Synthesis

**Figure 4:**
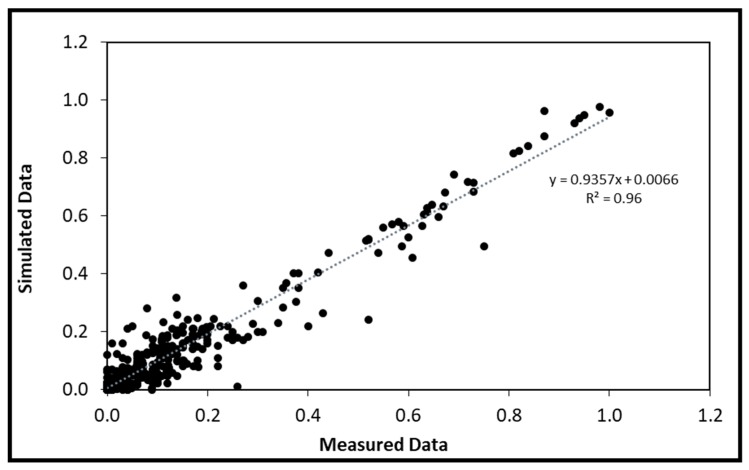
Comparison between predicted and measured data. The measured data correspond to the set of isotopologues of the three experimental conditions, which made it possible to calculate the flux data (*n* = 53) at 95% confidence interval and represent four biological replicates per experimental condition. These data are presented in detail in Supplementary Table S5. The linear regression equation and R2 value are shown.

13C-MFA allowed the quantification of metabolic fluxes during reserve lipid accumulation in flax embryos. The INCA software package was used for the calculations [39]. The program integrated: (i) The metabolic model (118 reactions and 12 pseudo-fluxes), (ii) the extracellular fluxes (substrate consumption and synthesis of storage products) and (iii) the experimental measures of the isotopic enrichments. Metabolic fluxes were obtained by minimizing the variance-weighted sum of squared residuals (SSR) between the experimentally measured and model predicted mass isotopologue distributions of 19 metabolites (free sugars, free organics acids, free amino acids and plastid acetyl-coA), with the measured external rates of three complementary labeling experiments (Cultures B–D) to the metabolic model. The three labeling experiments are culture B (containing a mix of glucose and glutamine 13C), culture C (containing unlabeled glucose and glutamine 13C) and culture D (containing glucose 13C and unlabeled glutamine). Thanks to the utilization of three parallel labeling experiments, estimated flux values were accurate and hence a precise carbon metabolic flux map was achieved [40]. Statistically acceptable fits were obtained; the minimized SSR values were lower than the maximum statistically acceptable SSR values at 95% confidence level. Comparisons between predicted and measured data are presented in Figure 4. The experimentally measured and simulated intracellular metabolite isotopologue are listed in Supplementary Table S5. The distribution is close to a linear relationship with a high correlation coefficient indicating that the mathematical flux model fits with the experimentally observed data.

**Figure 5:**
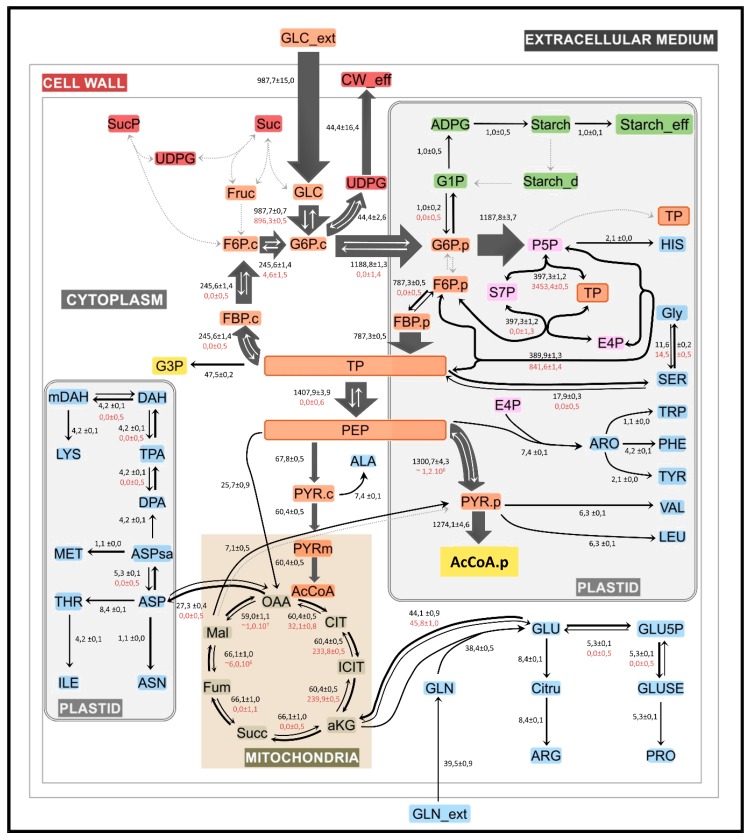
Metabolic network and fluxes in flax embryos during maturation stage. The scheme presented is a simplified representation of the central carbon metabolism in flax embryos during fatty acid synthesis and TAG accumulation. Cytosolic and plastidial metabolites are specified by subscripts c and p, respectively, if the model distinguishes such subcellular species. All reactions, metabolites and abbreviations are available in Supplementary Tables S3 and S4. Values reported are the best fit obtained by INCA software using three parallel labeling experiments (53 metabolites and six extracellular fluxes). The average and standard deviation are calculated from four independent experiments. Fluxes are expressed in mmol/gDW/d ± confidence interval. The set of estimated flux values calculated by INCA software are presented in Supplementary Table S6. The metabolic map was drawn using the Omix software [41].

The complete set of best-fit flux values, including 95% confidence intervals for all fluxes are also provided in Supplementary Table S6. The 13C-MFA results point out that glucose is the main source of carbon for synthesis and accumulation of storage lipids (TAG) during flax embryo development (Figure 5).

#### 2.5.2. Source of Cofactors for Storage Lipid Biosynthesis

There was high demand for the formation of lipids in the cofactors NADH and NADPH [42]; these cofactors and others were measured (Supplementary Table S3). For fatty acid synthesis, 1267 mmol NADPH/gDW/d, 1267 mmol NADH/gDW/d and 1267 mmol ATP/gDW/d were needed, and 47.5 mmol NADH/gDW/d and 47.5 mmol ATP/gDW/d for the synthesis of G3P. Moreover, the model identified the oxidative pentose pathway reactions glucose-6-phosphate dehydrogenase and 6-phosphogluconate dehydrogenase (Vppp) as the main sources of NADPH. These reactions generated 2374 mmol NADPH/gDW/d, 339 times more NADPH than that produced by the NADP-dependent malic enzyme (Vmal2). NADPH generated 1.9 times the demands required for fatty acid synthesis; hence, part of this pool contributes to the synthesis of amino acids within plastids (valine (VAL), leucine (LEU), proline (PRO), tryptophan (TRP), phenylalanine (PHE) and isoleucine (ILE)) and in the cytosol (proline (PRO) and glutamate (GLU)).

Another important cofactor for the synthesis of fatty acids and G3P is the NADH. The proposed model estimated that 1407 mmol NADH/gDW/d is required for TAG synthesis. Several reactions from different metabolic pathways produce NADH, although two of them are the main sources of this molecule, glyceraldehyde-3-phosphate dehydrogenase (Vgapdh: 1407 mmol NADH/gDW/d) and pyruvate dehydrogenase (Vpdhp: 1274 mmol NADH/gDW/d). Other reactions associated to the Krebs cycle and to the amino acid biosynthetic pathways produce 287 mmol NADH/gDW/d. In total, NADH produced, through these reactions, 2.1 times the necessities requested by the synthesis of fatty acids and TAG. NADH is also consumed by reactions generating ATP (Vnrj2). ATP also contributes to the synthesis of amino acids, fatty acids and TAG.

The model developed in this work predicted that 1.4 mmol of ATP/gDW/d is needed to support fatty acid and G3P synthesis in developing flax embryos. The proposed model considers six different ATP-generating reactions within three metabolic routes, energy reactions (Vnrj2, Vnrj3), glycolysis reactions (Vgapdh, Vpyrkp, Vpkc) and succinate biosynthetic pathway (Vnrj2). The reactions of glycolysis (Vgapdh, Vpyrkp) and energy (Vnrj2) mainly produce ATP, 1407, 1300, 1403 mmol ATP/gDW/d, whereas the reactions Vpkc (67.8 mmol ATP/gDW/d), Vkdh (66.1 mmol ATP/gDW/d) and Vnrj3 (66.1 mmol ATP/gDW/d) produce less. Global ATP production has been calculated to be 4310 mmol ATP/gDW/d, three times higher than the rate of ATP consumption during fatty acid and G3P synthesis; therefore, this production justifies the participation of this molecule in an array of metabolic processes (Supplementary Table S6).

## 3. Discussion

Flaxseed embryo oil is comprised of approximately 65% polyunsaturated ω-3 linolenic acid, making flax an important source of ω-3 fatty acids. Considerable efforts have been made in order to shed light on determining the route of carbon from central metabolism to de novo fatty acid synthesis in the embryos, with genome assembly [35], transcriptomics [11,36,37] and proteomics [21,22] approaches.

Pyruvate is the substrate of the pyruvate dehydrogenase complex that converts this glycolytic metabolite into plastidial acetyl-CoA. Acetyl-CoA carboxylase catalyses the first committed step of de novo fatty acid synthesis. As such, the identification of the main pathway contributing to the generation of the plastidial pyruvate pool is an important step toward a better understanding of the carbon flux from carbohydrates to lipids in flax metabolism. In this current work, the application of the state-of-the-art methods in 13C-MFA using a set of three parallel labeling experiments allowed the precise determination of metabolic fluxes, identification of the main carbon routes leading to oils and the principal sources of cofactors required by this metabolic process. Glucose imported from culture medium is converted to G6P in the cytosol, imported to plastids to feed the OPPP and enter into the plastidial glycolysis up to the formation of PYR.p. The synthesis of PYR.p can be carried out either from PEP by pyruvate kinase, or from malate by malic enzymes or by transporting PYR from the cytosol to the plastid. Like maize [26,43], sunflower [27], rapeseed [28,29] and *Arabidopsis thaliana* [30] embryos, PYR.p is mainly synthesized from PEP by pyruvate kinase in flax embryos. While this route is predominant, it is nevertheless higher in flax embryos (99.5%) compared to rapeseed embryos (74%), *Arabidopsis thaliana* (73%), sunflower (93%) and maize (between 70% and 54%) (Table 1).

In flax, rapeseed and *Arabidopsis thaliana*, the malic enzymes are not very active and generate, respectively, 0.5%, 0% and 2% of the PYR.p carbons, contrary to sunflower (7%) and maize (30–46%) (Table 1). However, differences between flax and rapeseed/*Arabidopsis thaliana* models remain. In rapeseed and *Arabidopsis thaliana* embryos, PYR.p is also derived from pyruvate imported from the cytosol to the plastid at 26% and 25%, respectively. In the case of flax, a pyruvate transporter from cytosol to plastid has not been identified by transcriptomics [11] or by proteomics [21,22] and was therefore not considered in the model developed herein. This transporter was also not taken into account in sunflower [27] and maize embryo [26,43] metabolic fluxes due to the lack of molecular evidence for such a carrier in plants [44].

Storage lipid biosynthesis requires a significant amount of energy, which is often limiting. This is particularly the case for NADPH and ATP. For flax, rapeseed, sunflower and maize, NADPH mainly comes from OPPP reactions and, to a lesser extent, from the NADP-dependent malic enzyme. Relative to demands of fatty acid synthesis, NADPH produced by OPPP reactions in flax embryos is in excess (187%) as observed for sunflower models (106%), while rapeseed (38%) and maize models (74–76%) do not fully meet the demand. For maize and sunflower models, NADP from malic enzymes is up to 30% and meets the demand. For Arabidopsis and rapeseed models, no data are available about NADPH needs.

ATP produced by TCA cycle activity via oxidative phosphorylation relative to biosynthetic demands is in excess for maize (200%) [26] and sunflower (>100%) [27] models but only partially in the arabidopsis (60%) [30], rapeseed (22%) [26,29,30] and flax (5%) models, indicating reduced TCA cycle activity in C3 metabolism. In the case of flax, rapeseed and arabidopsis, ATP production is provided by glycolysis reactions (Vgapdh, Vpyrkp, Vpkc) to form a pool necessary for storage lipid biosynthesis. Moreover, in flax seed, the energetic reaction allows the conversion of the surplus of NADPH and the use of ADP. This makes it possible to release a pool of NADH and ATP, ensuring the cell growth and the lipid storage biosynthesis. Finally, in this study, we deciphered flax metabolism during lipid storage biosynthesis. This new knowledge can be used in the future for metabolic engineering studies in order to identify novel biomarkers to enhance breeding of flax and yield improvements.

## 4. Materials and Methods

### 4.1. Chemicals and Solutions

[U-13C6] glucose, [2-13C1] glucose and [U-13C5] glutamine were purchased from Cambridge Isotope Laboratories. Standards, minerals, vitamins and other compounds were purchased from Sigma. Solvents for LC-HRMS were purchased from Biosolve BV. All media and stock solutions were sterilized by filtration.

### 4.2. Plant Material

Flax plant (*Linum usitatissimum* L.) line astral was kindly provided by Laboulet semence (Airaines, France). Flax plants were grown in a greenhouse by applying the recommendations described in reference [34]. Briefly, the plants were cultivated in a growth chamber at 60% relative humidity with a photoperiod of 16/8 h day/night cycle (20/16 °C) and a light intensity of 400 µmol photon m^−2^s^−1^. Flowers were tagged as petals opened. Flax seeds used for biochemical characterization were harvested at 14, 16, 18, 20, 22, 24, 26, 28, 30, 32, 34, 36, 38, 40, 43, 46 and 50 DAF immediately frozen in liquid nitrogen and stored at −80 °C. For embryo culture, capsules were harvested at 16 DAF, sterilized for 20 min in 50% (*v*/*v*) bleach and then rinsed for 20 min with autoclaved water, prior to embryo removal and desiccation.

### 4.3. In Vitro Embryo Culture

Ten aseptically dissected flax embryos (16 DAF) were transferred to a Petri dish containing 16 mL of an optimized culture medium. The culture medium reflected the composition of the embryo sac fluid [32,34]. It contained glucose (100 mM) and glutamine (800 mg/L) as macronutriments. It also included vitamins, minerals and micronutriments, KNO3 (125 mg/L), MgSO4.7H20 (370 mg/L), KCl (125 mg/L), KH2PO4 (10 mg/L), H3BO3 (10 mg/L), ZnSO4.7H20 (10 mg/L), Na2MoO4 (0.25 mg/L), CuSO4 (0.025 mg/L), CoCl2 (0.025) and nicotinic acid (5 mg/L). The pH was maintained at 8.0 and 20% (w/v) polyethylene 4000 provided the osmoticum. Four different cultures were made: One (A) containing unlabeled glucose and glutamine; a second (B) containing 20 mM [U-13C6] glucose and 160 mg/l [U-13C5] glutamine; a third (C) containing unlabeled glucose and 800 mg/L [U-13C5] glutamine; and a fourth (D) containing 60 mM [U-13C6] glucose with 40 mM [2-13C1] glucose and unlabeled glutamine. Cultures A and B were used during 24 h, 72 h, 120 h, 168 h, while culture C and D were used at 168 h of incubation. Four Petri dishes per point of kinetics and culture were made for optimal results. Cultures were performed in a growth chamber at 18 °C under a light intensity of 20 µE m^−2^s^−1^ with a humidity of 60%. Culture A was carried out to: i) Follow the evolution of the growth of embryos in vitro vs in planta; ii) determine the stationary metabolic state; iii) determine the rates of consumption of the substrates (glucose and glutamine) and the accumulation rates of the compounds of reserves (lipids, proteins, cell wall and starch). Culture B was used to determine the optimal incubation time for the stationary isotopic state to be reached necessary for 13C-MFA in stationary condition. The isotopic enrichments of the various metabolites from stationary isotopic B, C and D cultures were used as complementary parallel labeling experiments to determine the metabolic fluxes by simultaneous fit with the isotopic enrichment of all the metabolites of the three cultures with the mathematical model. For each culture, embryos were harvested and washed three times with 10 mL of water to remove surface labeling. The embryos were then placed in liquid nitrogen and lyophilized to give the dry weight, biomass accumulation and isotopic enrichment of metabolites.

### 4.4. Biomass Extraction and Quantification

The methods used for extraction, separation and quantifying biomass composition are described in reference [26,27,45]. For this, lipids, protein, starch and cell wall were extracted successively from 10 mg of lyophilized flax embryos.

#### 4.4.1. Lipids and Fatty Acid Analysis

Lipids were extracted from flax seeds and flax embryos as described in reference [34]. Briefly, flax seeds or flax embryos were ground using a Precellys 24-Dual homogenizer for 5 min at 7.3 g in the presence of glass beads (0.5 mm) and 1 mL of hexane/isopropanol (2:1). After a centrifugation for 5 min at 16,000× *g*, the upper phase was collected. The operation was repeated twice on the pellet to optimize lipid extraction. The three supernatants were then transferred to a previously weighed glass tube and the biomass pellet was dried at 40 °C. Total lipid content was then determined gravimetrically after hexane evaporation under nitrogen. Lipids were derivatized by transesterification to generate fatty acid methyl esters (FAMEs) using tetramethylammonium hydroxide (TMAH). For this, 5 mg of total lipid was resuspended in 100 μL of diethyl ether and was transesterified by the addition of 5 μL of TMAH. The reaction was carried out at 25 °C for 15 min with stirring at 625 rpm and then stopped by the addition of 50 μL of decane. After centrifugation for 5 min at 16.1 g, 20 μL of the upper phase were taken and diluted in 178 μL of heptane to be analyzed by GC-MS.

FAMEs were analyzed using a ThermoFisher GC–MS (DSQ II) instrument equipped with a BPX70 capillary column (60 m × 0.25 mm; 0.25 μm) from SGE analytical science. FAME derivatives were separated by a gradient of temperature and by using helium as the vector gas at a constant flow rate of 0.9 mL/min. The GC oven temperature was set at 140 °C and held for 2 min. The oven temperature was then increased to 19 °C/min to reach a final temperature of 230 °C which was held for 2 min. The injection temperature was set at 230 °C with a splitless mode. MS analysis was realized using electron impact (EI) ionization in positive ion mode. The ion source and interface temperatures were set at 230 °C. Detection was in full scan 217 mode between *m*/*z* 50 and 500. GC-MS data were acquired and processed using Xcalibur software. FAME derivatives were identified using the NIST library and neat FAME standards were purchased from Sigma.

#### 4.4.2. Proteins and Amino Acid Concentration

The dried pellet obtained in the previous step was taken up in 1 mL of 20 mM Tris-HCl extraction buffer, pH 7.5, 150 mM NaCl and 1% SDS as described in reference [26,27,45]. After incubation for 15 min at 42 °C in a shaker at 0.2 g, the samples were centrifuged for 15 min at 16,000× *g*. The supernatant was recovered and the operation was repeated twice. The protein extract was then diluted to the tenth and 100 μL was then taken for protein quantification. Quantification of the soluble proteins present in our samples is based on the Lowry’s method [46]; 125 μL of a solution of BIO-RAD copper tartrate alkaline buffer was added. After 10 min, 1 mL of Lowry’s reagent was added, and the absorbance was read at 750 nm. Protein concentrations were determined using a standard range made with BSA (bovine serum albumin) from 0 to 1.5 mg/mL. The pellet was dried again.

Extraction of the amino acids from the extracted soluble proteins was carried out by acid hydrolysis of the proteins. For this, the soluble proteins were first precipitated by adding 200 μL of trifluoroacetic acid. After 1.5 h of incubation on ice, the samples were centrifuged for 20 min at 16,000× *g* and 250 µg/mL of [U-13C5] glutamine as the internal standard was added to the supernatant. The pellet was taken up in 200 μL of HCl (6N). A 24 h incubation at 110 °C, 200 μL of 6N NaOH was added. After centrifugation for 10 min at 16,000× *g*, the supernatants were recovered for injection into LC-HRMS. Quantification of the amino acids was performed using ultra high-pressure liquid chromatography (UPLC 1290 Infinity) coupled with high resolution–mass spectrometry (HRMS Q-TOF UHD 6538) from Agilent Technologies. The total LC-HRMS run was 30 min with a flow rate of 0.25 mL/min. Amino acids were separated on a Thermo Hypersyl Gold Phenyl (150 × 2.1 mm, 3 μm) at 20 °C, using an elution gradient composed of a solution of 5 mM anhydrous pentafluoropropionic acid in water (solvent A) and acetonitrile (solvent B). The gradient was as follows: A = 0–4 min 99%, 4–12 min 12%, 12–15 min 70%, 15–30 min 2%. The mass spectra were acquired using a dual electrospray ionization in positive-ion mode. The source temperature, fragmentor and the skimmer were set up, respectively, at 350 °C, 120 V, 65 V. The acquisition was made in full scan mode between 50 *m*/*z* and 1050 *m*/*z*, with a scan of two spectra per second. MassHunter B.07 software, allowed to control the parameters of the machine, acquired and processed the data. Amino acid quantification was performed by injection of amino acid standards between 20 μg/mL and 500 μg/mL and contained 250 µg/mL of [U-13C5] glutamine as internal standard.

#### 4.4.3. Starch and Cell Wall

Extraction and quantification of the starch was achieved using a Total Starch Assay Kit (AA/AMG) from Megazyme International Ireland Ltd. Starch extraction was performed on the pellets after protein extraction. The remaining metabolites, minerals and vitamins were removed by washing the pellets three times with water and three times with ethanol. Samples were taken up in 1 mL of water, stirred for 5 min in a shaker at 2.3 g and the supernatant was removed. This washing was done three times with water and three times with ethanol. The pellet obtained was taken up in 600 μL of a solution of α-amylase with 250 µg/mL of [U-13C6] glucose as the internal standard (sodium acetate pH 4.8, α-amylase 1600 U/mL (30/1), (*v/v*)) and incubated at 100 °C for 30 min. Once the mixture cooled down to room temperature, 20 μL of α-amyloglucosidase (200 μM) were added, and samples were incubated for 30 min at 50 °C. After centrifugation (5 min at 16,000× *g*), a 500 µL aliquot of supernatant was pipetted into a 1.5 mL tube to be used for quantification by LC-HRMS. Separation conditions were performed as described in reference [34]. Quantification was performed by injecting glucose standards between 20 μM and 500 μM containing 250 µg/mL of [U-13C6] glucose as the internal standard. Total cell wall content was then determined gravimetrically after the pellet was successively washed (three times with water, three times with 70% ethanol and three times with acetone) and dried for 72 h under the fumehood as described in references [26,27,45].

### 4.5. Metabolites Extraction

Water-soluble metabolites (free sugars, free amino acids and organic acid) were extracted from 10 mg of dried flax embryos as described in reference [35]. Briefly, after extracting lipids as described previously, the pellet was taken up in 1 mL of boiling 0.01% HCl and placed in a water bath at 95 °C for 15 min. After centrifugation for 10 min at 16,000× *g*, the supernatants were filtered (0.22 μm) and collected. This procedure was repeated three times, to maximize metabolite extraction. Supernatants were frozen, freeze-dried and taken up in 1 mL of sodium acetate buffer (50 mM, pH 5.5). Samples were loaded onto a cation exchange resin (Dowex 50 × 8 (hydrogen form, 200–400)) to collect free amino acid fractions. The recovered fraction containing the free sugars and organic acids were loaded onto an anion exchange resin (Dowex 1 × 8 (formate form, 200–400, Supelco 13858)). Free sugars were collected after an elution with 5 mL of water, whereas organic acids were eluted using formic acid (4 M). The three fractions (amino acids, organic acids, free sugars) were frozen and lyophilized to be taken up in 500 μL of water for the determination of the isotopic enrichment of these molecules.

### 4.6. Isotopic Enrichment of Metabolites

Isotopic enrichments were determined on FAMEs, and water-soluble metabolites (free sugars, free amino acids and organic acid). FAMEs were separated and analyzed as previously described. Labeling of FAMEs was determined on the base peak of methyl palmitate. This peak corresponds to the McLafferty rearrangement fragment (*m*/*z* 74). It has two carbons from plastidial acetyl CoA (AcCoA.p). Labeling of free sugars and free amino acids was determined as described in references [32,34,47].

Organic acids were separated using ultra-high-pressure liquid chromatography (UPLC 1290 Infinity) coupled with high resolution–mass spectrometry (HRMS Q-TOF UHD 6538) from Agilent Technologies. Organic acids were separated on a Dionex Acclaim OA column (150 × 2.1 mm, 3 μm), at a flow rate of 0.5 mL/min with isocratic condition (0.2% formic acid in water). The total LC-HRMS run was 5 min. At the end of the chromatographic column, the eluted molecules were identified by mass spectrometry. Organic acids were ionized in negative mode using a dual electrospray ionization at 350 °C with a fragment of 120 V and a value of 65 V Skimmer. Organic acids were detected in full scan mode using a mass range from 100 *m*/*z* to 3000 *m*/*z*. Organic acids were identified by finding the exact mass and retention time of the succinate, fumarate, citrate/isocitrate and malate standards. Mass isotopologue distributions were obtained by integration of isotope mass from base peaks [M-H+], and corrected for natural abundances using Scilab.

### 4.7. Modeling Metabolic Pathways

A central carbon metabolic network model of flax embryos was constructed for 13C-MFA analysis. The metabolic model described in Supplementary Figure S1 and Supplementary Table S3 was programmed in the INCA software [39] based on the elementary metabolite unit decomposition for the underlying isotopologue network (EMU). Intracellular fluxes were estimated by the best fit of three complementary labeling experiments in parallel (mass isotopologue distributions of 53 intracellular metabolites and six extracellular fluxes measured of Cultures B–D) with the model predicted to be a single flux model. As initial program, 2000 different starting points (random initial values), using a Levenberg–Marquardt optimization algorithm [48], were used. After fixing the best value, an optimization program was performed with 300 starting points. Accurate 95% confidence intervals were computed for all estimated fluxes by evaluating the sensitivity of the minimized SSR to flux variations. Least-squares parameter regression and statistical and sensitivity analyses of the optimal solution were performed using the INCA software [39]. An χ^2^ statistical test to assess goodness of fit and accurate 95% confidence intervals were computed by evaluating the sensitivity of the minimized SSR to flux variations. Assuming that the model is correct when the acceptable range of SSR values is between 95% confidence interval [49]. According to [43], a linear regression of a comparison between mass isotopologue distribution measured and simulated (Figure 4 and Supplementary Table S5) with a coefficient of variation close to 1 allows the model to be validated. The metabolic map was drawn using Omix Software [41]. The results are presented in Figure 5 and Supplementary Table S6.

## Figures and Tables

**Table 1:** Comparison of carbon source for PYR.p and NADPH between flax, rapeseed, *Arabidopsis thaliana*, sunflower and maize cultivated developing embryos during lipid biosynthesis and accumulation calculated from 13C-MFA analysis.

	Reactions	Flax Embryos Astral	Rapeseed Embryos Reston [25,28,29]	*A. thaliana* Embryos ws, pkp, col, wri [30]	Sunflower Embryos Ames 7576 [27]	Maïze Embryos LH 59 [28]	Maïze Embryos Alex [43]
**Carbon source for PYR.p (mol%)**	PEP -> PYR.p	99.50%	74%	73% to 88%	93%	70%	54%
	MAL -> PYR.p	0.50%	NC	2%	7%	30%	46%
	PYR.c -> PYR.p	NC	26%	10% to 25%	NC	NC	NC
**Source of NADPH relative to demand for fatty acids biosynthesis (mol%)**	by OPPP	187%	38%	-	106%	76%	74%
	by malic enzyme	0.55%	NC	-	7%	30%	56%

## Supplementary Materials

The following are available online at https://www.mdpi.com/2218-1989/10/1/14/s1, Figure S1: The metabolic network of flax embryos cell, Table S1: The amino acid composition of proteins, Table S2: Evolution of the isotopic enrichment of free organic acids and two carbons from palmitic acid (C16:0) extracted from developing embryos harvested at 16DAF incubated during 24 h, 72 h, 120 h, 168 h in the culture medium B, containing 80 mM unlabeled glucose, 20 mM [U-13C] glucose, 640 mg/L unlabeled glutamine, 160 mg/L [U-13C] glutamine, Table S3: Lists of reactions used with carbon transition of the metabolic network of flax embryos cell, Table S4: Lists of metabolites and details of their abbreviations used in the metabolic network of flax embryos cell., Table S5: Comparison between mass isotopomer distribution measured and simulated, Table S6: Metabolic fluxes in flax embryos during lipids accumulation.
