# Supplementary material for: 13C-Metabolic Flux Analysis in Developing Flax (Linum usitatissinum L.) Embryos to Understand Storage Lipid Biosynthesis
Source: Metabolites. 2019 Dec 24;10(1):14. doi: 10.3390/metabo10010014 (PMC7022742; doi:10.3390/metabo10010014)
Supplement: Supplementary file 1 [file metabolites-10-00014-s001.zip › Supplementary Figure S1.pdf]

13C-Metabolic Flux Analysis in Developing Flax (Linum usitatissinum L.) Embryos to Understand Storage Lipid Biosynthesis

Sébastien Acket [1] *, Anthony Degournay [1], Yannick Rossez [1], Stéphane Mottelet [2], Pierre Villon [3], Adrian Troncoso-Ponce [1], and Brigitte Thomasset [1]

[1] Alliance Sorbonne Université, Génie Enzymatique et Cellulaire, UMR CNRS 7025, Université de Technologie de Compiègne, 60205 Compiègne Cedex, France.

[2] Alliance Sorbonne Université, EA 4297 TIMR, Transformations Intégrées de la Matière Renouvelable, Université de Technologie de Compiègne, 60205 Compiègne Cedex, France.

[3] Alliance Sorbonne Université, Laboratoire Roberval, FRE UTC CNRS 2012, Université de Technologie de Compiègne, 60205 Compiègne Cedex, France.

* Corresponding author. Email address: sebastien.acket@utc.fr

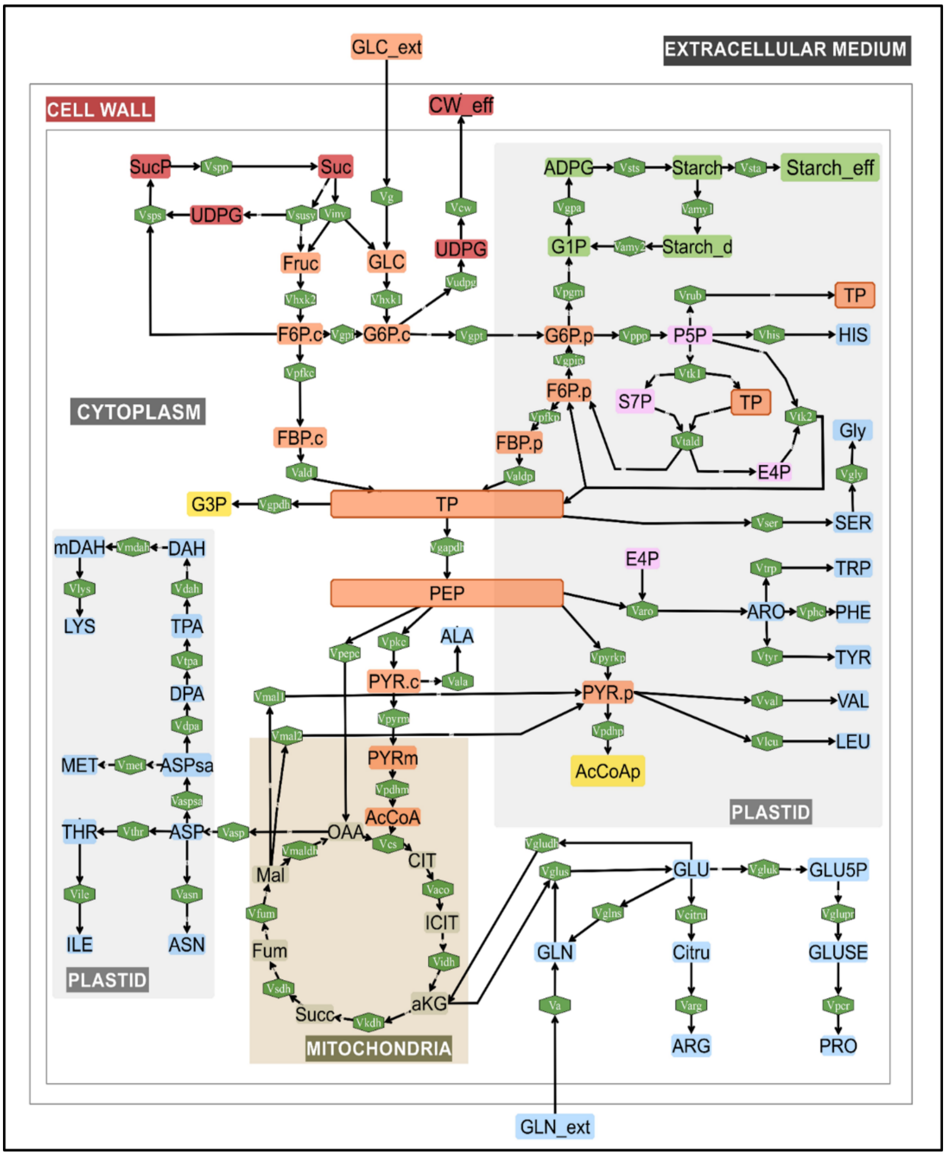

Supplementary Figure S1: The metabolic network of flaxseed embryos cell.

The illustration was designed with Omix [41].

[41] Droste, P.; Nöh, K.; Wiechert, W. Omix – A Visualization Tool for Metabolic Networks with Highest Usability and Customizability in Focus. *Chemie Ingenieur Technik* **2013**, *85*, 849–862.
The reactions are detailed in the Supplementary Table S3. Abbreviations of reactions and metabolites are respectively detailed in the Supplementary Table S3 and S4. For better clarity, the cofactors, and the reaction of protein and triacylglyceride synthesis have not been modeled. You will find the details of these reactions in the Supplementary Table S3 and S4.
